# Risk Factor for Retreatment Episode on Admission Among TB Patients With Schizophrenia

**DOI:** 10.3389/fpsyt.2021.793470

**Published:** 2021-12-09

**Authors:** Hai-Rong Wang, Chao Han, Jun-Li Wang, Yan-An Zhang, Mao-Shui Wang

**Affiliations:** ^1^Department of Outpatient, Shandong Mental Health Center, Jinan, China; ^2^Department of Lab Medicine, The Affiliated Hospital of Youjiang Medical University for Nationalities, Baise, China; ^3^Department of Cardiovascular Surgery, Shandong Public Health Clinical Center, Cheeloo College of Medicine, Shandong University, Jinan, China; ^4^Shandong Key Laboratory of Infectious Respiratory Disease, Jinan, China; ^5^Department of Lab Medicine, Shandong Provincial Chest Hospital, Cheeloo College of Medicine, Shandong University, Jinan, China; ^6^Department of Lab Medicine, Shandong Public Health Clinical Center, Cheeloo College of Medicine, Shandong University, Jinan, China

**Keywords:** tuberculosis, schizophrenia, retreatment, outcome, risk factor

## Abstract

**Background:** The clinical characteristics of patients with tuberculosis (TB) and schizophrenia remain largely unknown. Furthermore, TB retreatment is associated with a poor outcome. Hence, we aimed to address the risk factors of TB retreatment in schizophrenia patients in this retrospective cohort.

**Methods:** Between March 2005 and August 2020, patients diagnosed with schizophrenia and TB were included in the study. Patient characteristics, such as demographics, medical history, underlying diseases, symptoms, outcome, and lab examinations, were collected from medical records using a structured questionnaire. TB retreatment was defined as treatment failures and relapses. Subsequently, multivariate logistic regression was performed using variables selected based on prior findings as well as factors found to be associated with a retreatment episode in univariate analyses (*p* < 0.1).

**Results:** A total of 113 TB patients with schizophrenia were included. Of them, 94 (83.2%) patients were classified as initial treatment group, and 19 (16.8%) were classified as retreatment group. The mean age was 53.0 ± 23.2 years, and males accounted for 61.9% of all cases. Multivariate analysis revealed that continuous antipsychotics treatment (OR = 0.226, 95% CI: 0.074, 0.693; *p* = 0.009) and extra-pulmonary TB (OR = 0.249, 95% CI: 0.080, 0.783; *p* = 0.017) were associated with the retreatment in TB patients with schizophrenia.

**Conclusion:** Retreatment is a significant concern for TB patients with schizophrenia. To improve the current dilemma, continuous antipsychotics treatment is required, and increasing awareness of schizophrenia would reduce the disease burden.

## Introduction

Tuberculosis (TB) a public health problem worldwide. In 2020, 9.9 million people are estimated to fall ill with TB (1). Unfortunately, schizophrenia makes the situation more complicated. First, a higher incidence of TB was reported in patients with schizophrenia, compared with the corresponding age groups in the general population (2). Second, most sputum collected from schizophrenia patients were salivary, which were insufficient for microscopy and culture (3). Third, a higher rate (63.9%) of adverse reactions is revealed in schizophrenics receiving anti-TB therapy (4).

Similarly, TB also confuses the situation of schizophrenia. For example, a meta-analysis demonstrated that the prevalence of TB in the schizophrenia population was 0.3% (95% CI 0.1–0.8) (5). Historically, TB was known as one of the commonest causes of death in schizophrenia patients (6). The drug interaction could reduce the effectiveness of antipsychotic drugs (7). Trifluoperazine, an antipsychotic drug approved by the Food and Drug Administration, effectively reduces cerebral edema by regulation of aquaporin-4 (8, 9). In addition, TB was also significantly associated with low physical activity (10).

To date, the clinical characteristics of patients with TB and schizophrenia remain largely unknown. Therefore, this retrospective cohort was performed aiming to describe it fully. As known, TB retreatment is associated with a poor outcome (11, 12). Our previous data showed that TB retreatment as a treatment outcome accounted for 33.3% of schizophrenia patients (13). Hence, as one aim of the cohort, the risk factors of TB retreatment in schizophrenia patients would be addressed in the current study.

## Methods

### Ethics

The study protocol was approved by the Ethics Committee of Shandong Provincial Chest Hospital and was conducted retrospectively at the hospital in accordance with the Declaration of Helsinki. Due to the retrospective nature and anonymous data collection, written informed consent was waived by the Ethics Committee of Shandong Provincial Chest Hospital.

### Subjects

Patients included in the study were diagnosed with the following: (1) schizophrenia according to Diagnostic and Statistical Manual of Mental Disorders, Fourth Edition, Text Revision (DSM-IV-TR) criteria or International Classification of Diseases, 10th revision; (2) TB according to WHO criteria (14), including evaluation with chest X-rays, symptoms, microscopy, mycobacterial culture, and real-time polymerase chain reaction (PCR). Patient characteristics, such as demographics, medical history, underlying diseases, symptoms, outcome, and lab examinations, were collected from medical records using a structured questionnaire.

### Definition

TB retreatment is defined as meeting the following criteria: (1) treatment failures—patients made unscheduled hospital readmission after ≥1 month of anti-TB therapy and (2) relapses—patients completed treatment successfully and were again diagnosed with TB (15). Pulmonary TB is defined as a case with TB affecting the lungs (which excludes the pleura and intrathoracic lymph nodes). Extrapulmonary TB is defined as TB with non-pulmonary presentations, and including pleural, lymphatic, osseous, meningeal, peritoneal, and other TB. Duration of schizophrenia is defined as the time since the first diagnosis. The concealment was recorded if no past medical history of schizophrenia was reported during the preliminary visit. Continuous antipsychotics treatment is defined as routine medication use from the time of schizophrenia diagnosis to TB diagnosis. Liver function impairment is defined as an increase in the aspartate transaminase and/or alanine transaminase level of at least twice higher than the upper limit of normal. Acid-fast bacilli (AFB) smear and mycobacterial culture were performed using auramine–rhodamine staining and Löwenstein–Jensen (LJ) medium, respectively. Hospital stay was defined as the number of days from admission to discharge or death of patients.

### Statistical Analysis

Data were analyzed with SPSS 16.0 (SPSS Inc., Chicago, USA). Differences between retreatment and initial treatment groups were compared using the chi-square test (or Fisher's exact) for categorical variables and *t*-tests for continuous variables. In multivariate logistic regression, variables selected based on prior findings as well as factors found to be associated with a retreatment episode in univariate analyses (*p* < 0.1) were included in multivariable logistic regression models. The odds ratio and 95% confidence interval were then calculated. Significance was considered if *p*-value was < 0.05.

## Results

### Baseline Characteristics

Between March 2005 and August 2020, a total of 113 TB patients with schizophrenia were included. Of them, 94 (83.2%) patients were classified as initial treatment group, and 19 (16.8%) were classified as retreatment group (*n* = 5, readmission; *n* = 14, recurrence). The mean age was 53.0 ± 23.2 years, and males accounted for 61.9% of all cases. TB contact history was reported in 12 patients, 91 patients underwent continuous antipsychotics treatment, and 33 patients were transferred from mental health services directly. The mean duration of schizophrenia was 15.1 ± 11.1 years. Table 1 shows the characteristics of TB patients with schizophrenia.

**Table 1:** The characteristics of schizophrenia patients with initial treatment and retreatment TB.

**Variables**	**Total**	**Initial treatment TB**	**Retreatment TB**	***P* value**	**Odds ratio**
*N*	113	94	19		
**Demographics**					
Sex, male	70 (61.9%)	59 (62.8%)	11 (57.9%)	0.69	
Age (years)	53.0 ± 23.2	51.9 ± 22.1	58.1 ± 28.0	0.292	
Urban area	67 (59.3%)	55 (58.5%)	12 (63.2%)	0.707	
**Medical history**					
TB contact	12 (10.6%)	9 (9.6%)	3 (15.8%)	0.428	
Smoking habit (package-years)	6.2 ± 15.5	5.6 ± 15.2	9.4 ± 16.7	0.333	
Duration of schizophrenia (years)	15.1 ± 11.1	15.2 ± 10.6	14.4 ± 13.6	0.903	
Patient concealment of schizophrenia	6 (5.3%)	6 (6.4%)	0 (0.0%)	0.999	
Alcohol use	11 (9.7%)	10 (10.6%)	1 (5.3%)	0.481	
Transferred from mental health services	33 (29.2%)	32 (34.0%)	1 (5.3%)	0.034	0.108 (0.014, 0.843)
Continuous antipsychotics treatment	91 (80.5%)	81 (86.2%)	10 (52.6%)	0.002	0.178 (0.061, 0.522)
**TB presentations**					
Pulmonary TB	86 (76.1%)	68 (72.3%)	18 (94.7%)	0.067	
Miliary TB	3 (2.7%)	2 (2.1%)	1 (5.3%)	0.453	
Extrapulmonary TB	65 (57.5%)	60 (63.8%)	5 (26.3%)	0.005	0.202 (0.067, 0.611)
Tuberculous pleural effusion	45 (39.8%)	42 (44.7%)	3 (15.8%)	0.027	0.232 (0.063, 0.850)
Tuberculous peritonitis	11 (9.7%)	11 (11.7%)	0 (0.0%)	0.999	
Lymph node TB	10 (8.8%)	9 (9.6%)	1 (5.3%)	0.552	
Osseous TB	9 (8.0%)	8 (8.5%)	1 (5.3%)	0.637	
Tuberculous meningitis	5 (4.4%)	5 (5.3%)	0 (0.0%)	0.999	
**Comorbidities**					
Diabetes mellitus	22 (19.5%)	19 (20.2%)	3 (15.8%)	0.658	
Cardiovascular diseases	16 (14.2%)	14 (14.9%)	2 (10.5%)	0.62	
Hypertension	12 (10.6%)	12 (12.8%)	0 (0.0%)	0.999	
Liver function impairment	10 (8.8%)	9 (9.6%)	1 (5.3%)	0.552	
**Symptoms**					
Cough	68 (60.2%)	54 (57.4%)	14 (73.7%)	0.194	
Sputum production	47 (41.6%)	36 (38.3%)	11 (57.9%)	0.119	
Fever	44 (38.9%)	38 (40.4%)	6 (31.6%)	0.472	
Chest tightness	27 (23.9%)	21 (22.3%)	6 (31.6%)	0.392	
Dyspnea	11 (9.7%)	11 (11.7%)	0 (0.0%)	0.999	
Chest pain	11 (9.7%)	7 (7.4%)	4 (21.1%)	0.081	
Hemoptysis	5 (4.4%)	2 (2.1%)	3 (15.8%)	0.024	8.625 (1.334, 55.755)
**Treatment history**					
Self-treatment	17 (15.0%)	11 (11.7%)	6 (31.6%)	0.034	3.483 (1.099, 11.040)
Outpatient treatment	70 (61.9%)	56 (59.6%)	14 (73.7%)	0.253	
Inpatient treatment	42 (37.2%)	35 (37.2%)	7 (36.8%)	0.974	
Hospital stay (days)	30.5 ± 33.3	31.4 ± 35.4	25.9 ± 19.9	0.516	
Death	5 (4.4%)	4 (4.3%)	1 (5.3%)	0.846	
**Radiological findings**					
Cavity	28 (24.8%)	22 (23.4%)	6 (31.6%)	0.454	
Calcification	12 (10.6%)	10 (10.6%)	2 (10.5%)	0.988	
**TB assays (+)**					
Smear	26 (26/104, 25.0%)	20 (20/85, 23.5%)	6(6/19, 31.6%)	0.519	
Culture	41(41/105, 39.0%)	35(35/87, 40.2%)	6(6/18, 33.3%)	0.784	
PCR	23 (23/68, 33.8%)	17(17/54, 31.5%)	6(6/14, 42.9%)	0.493	
IGRA or PPD	47 (47/58, 81.0%)	41 (41/51, 80.4%)	6(6/7, 85.7%)	0.051	
**Lab examinations**					
WBC (10^9^/L)	7.26 ± 3.10	7.19 ± 3.11	7.59 ± 3.13	0.523	
Neutrophil (10^9^/L)	5.2 ± 2.9	5.3 ± 2.9	5.0 ± 2.9	0.78	
Lymphocyte (10^9^/L)	1.3 ± 0.7	1.3 ± 0.6	1.6 ± 0.8	0.065	
Moncyte (10^9^/L)	0.6 ± 0.3	0.6 ± 0.3	0.7 ± 0.5	0.09	
Esophil (10^9^/L)	0.09 ± 0.11	0.08 ± 0.10	0.14 ± 0.13	0.825	
Basophil (10^9^/L)	0.01 ± 0.01	0.01 ± 0.01	0.01 ± 0.01	0.19	
Erythrocyte sedimentation rate (mm/h)	43.1 ± 31.6	44.9 ± 31.5	34.6 ± 31.4	0.215	
Total protein (g/L)	66.2 ± 8.1	66.1 ± 8.2	66.7 ± 7.3	0.03	0.978 (0.959, 0.998)
Albumin (g/L)	35.2 ± 6.0	34.8 ± 5.7	38.3 ± 7.0	0.132	

*TB, tuberculosis; PCR, polymerase chain reaction; IGRA, interferon gamma release assay; PPD, purified protein derivative; WBC, white blood cell*.

TB cases included pulmonary TB (*n* = 86, 76.1%) and extrapulmonary TB (*n* = 65, 57.5%). The presentation of extrapulmonary TB included tuberculous pleural effusion (*n* = 45, 39.8%), tuberculous peritonitis (*n* = 11, 9.7%), lymph node TB (*n* = 10, 8.8%), osseous TB (*n* = 9, 8.0%), tuberculous meningitis (*n* = 5, 4.4%), and miliary TB (*n* = 3, 2.7%). Comorbidities were as follows: diabetes mellitus (*n* = 22, 19.5%), cardiovascular diseases (*n* = 16, 14.2%), hypertension (*n* = 12, 10.6%), and liver function impairment (*n* = 10, 8.8%).

Cough was the most common symptom, followed by sputum production (*n* = 68, 60.2%), fever (*n* = 47, 41.6%), chest tightness (*n* = 44, 38.9%), dyspnea (*n* = 11, 9.7%), chest pain (*n* = 11, 9.7%), and hemoptysis (*n* = 5, 4.4%). The mean hospital stay was 30.5 ± 33.3 days. Self-treatment, outpatient treatment, and inpatient treatment were reported in 17 (15.0%), 70 (61.9%), and 42 (37.2%) patients, respectively. In addition, there were 5 (4.4%) deaths during hospitalization.

The positive rates of microbiological examination were calculated as follows: AFB smear (25.0%, 26/104), culture (39.0%, 41/105), TB PCR (33.8%, 23/68), and interferon-gamma release assay (IGRA) or purified protein derivative (PPD) test (81.0%, 47/58). Other lab examinations, such as blood cell count and chemistry analysis, are listed partially as in Table 1.

### Univariate and Multivariate Analysis

Table 1 also shows the results of univariate analysis by a comparison between initial treatment and retreatment groups. It was found that the retreatment episode was associated with transferring from mental health services, continuous antipsychotics treatment, extra-pulmonary TB, tuberculous pleural effusion, hemoptysis, self-treatment, and total protein (all *p* < 0.05).

Further multivariate analysis (Hosmer–Lemeshow goodness-of-fit test: χ^2^ = 0.770, *df* = 2, *p* = 0.681) revealed that continuous antipsychotics treatment (OR = 0.226, 95% CI: 0.074, 0.693; *p* = 0.009) and extra-pulmonary TB (OR = 0.249, 95% CI: 0.080, 0.783; *p* = 0.017) were associated with retreatment in TB patients with schizophrenia (Table 2).

**Table 2:** Multiple logistic regression analysis of risk factors for retreatment in TB patients with schizophrenia.

**Variables**	***P* values**	**OR (95%)**
Continuous antipsychotics treatment	0.009	0.226 (0.074, 0.693)
Extrapulmonary TB	0.017	0.249 (0.080, 0.783)

*TB, tuberculosis; OR, odds ratio*.

## Discussion

Currently, TB retreatment is still a serious threat for TB control. Its treatment success remains relatively low, and a tailored drug regime is required (16, 17). Unfortunately, schizophrenia occurs with TB and makes the management of TB retreatment more complex. It is recommended that the risk factors for retreatment cases should be evaluated individually, and then specific strategies are required to improve the outcome of TB retreatment (18). Hence, this retrospective study was conducted, and several interesting findings were observed in patients with TB and schizophrenia. In our study, continuous antipsychotics treatment and extra-pulmonary TB were identified as two key protective factors for retreatment episodes. To our knowledge, our study is the first to investigate the corresponding risk factors during the past years.

TB retreatment accounted for a high proportion (16.8%) of TB patients with schizophrenia, which is higher than that of general TB patients (13%) in our center (19). However, when taking it as a treatment outcome of our study, the rate would increase to approximately one-third of the population (13). The difference may be explained in that most cases who experience readmission treatment were only reported as cured cases due to recall bias. The risk factors of retreatment among TB patients have been well-characterized, and several factors have been identified previously, such as age, cavitation, smoking, educational status, and alcohol use (18, 20, 21). In general, retreatment patients have a lower cure rate than initial treatment TB and encounter more side effects during treatment with second-line drugs (22). The associations between these variables and retreatment status were mainly explained by three key factors: (1) drug resistance was associated with TB treatment failure and relapse (23); besides, acquisition of drug resistance was common during failure of empiric drug regimens for TB (24); (2) treatment interruption is common for TB retreatment (25); and (3) due to a high mortality rate, retreatment status denotes a serious medical condition (11, 26, 27).

In our study, continuous antipsychotics use is identified as a protective factor for TB retreatment. As known, due to inadequate self-management, the schizophrenia condition could lead to poor medications for other chronic illnesses, such as diabetes and hypertension (28–30). Meanwhile, several studies have investigated the role of antipsychotics use in the management of schizophrenia patients with comorbid chronic illness. Antipsychotic adherence is associated with improved diabetes outcomes among individuals with schizophrenia (31). In addition, continuous antipsychotics use could lower the utilization of medical emergency department in patients with schizophrenia (32). Similarly, our study also demonstrated an association between antipsychotic adherence and poor TB outcome (retreatment). This should be explained by the fact that treatment interruption is common in schizophrenia patients with poor antipsychotic adherence. This point has been proved by our previous study (13). Similarly, antipsychotics use is found to be associated with adherence to cardiometabolic medications in patients with schizophrenia (33).

Extrapulmonary TB is another protective factor for retreatment episode. This may be related to the fact that most extrapulmonary TB cases require special surgical techniques or nursing attention. In our study, 39.8% of enrolled patients had tuberculous pleural effusion; these patients required thoracentesis to relieve dyspnea or make a diagnosis. This finding was in agreement with previous studies. Dangisso et al. found that the treatment success was higher for extrapulmonary TB compared to pulmonary TB (34). Non-recurrent cases had a significantly high fraction of extrapulmonary TB (recurrent: 0.0%, non-recurrent: 13.4%; *p* = 0.036) (35). In addition, in Chinese recurrent TB cases, relapse is more common than reinfection (36). Similar findings were revealed by Shen et al. (37) and Qiu et al. (38). Therefore, it was guessed that in our study, most cases may experience relapse. This point also supports extrapulmonary TB as a protective factor for retreatment episodes. Nevertheless, in most studies, it is thought that extrapulmonary TB is an independent risk factor for unfavorable events (39–42). The disagreement may be attributed to more care given to these patients and the rarity of severe forms of extrapulmonary TB [such as miliary TB (2.1 vs. 6.6%) and tuberculous meningitis (5.3 vs. 6.8%)], in comparison with data from Pang et al. (43).

Our study has several limitations. First, due to the retrospective nature, selection bias remains a concern. Second, the study has a long observation period, which could lead to a significant effect on the results due to improvement in medical care. Third, due to secondary data limitations, incomplete data are frequently encountered and would result in a substantial loss in precision and power, which adversely affects the data usefulness. Fourth, to achieve statistical significance, patients were not further classified to readmission and recurrent cases. Additionally, to ensure the comparability of baseline characteristics, only new cases on the first admission were recruited in our study.

## Conclusions

TB retreatment is a significant concern for TB patients with schizophrenia; continuous antipsychotics treatment and extrapulmonary TB were found to be protective against retreatment episode. To improve the status of TB retreatment, increasing awareness of schizophrenia is required, and effective management of schizophrenia would reduce the disease severity.

## Data Availability Statement

The original contributions presented in the study are included in the article/supplementary material, further inquiries can be directed to the corresponding author/s.

## Ethics Statement

This study was conducted at the Shandong Provincial Chest Hospital in accordance with the Declaration of Helsinki. The study protocol was approved by the Ethics Committee of Shandong Provincial Chest Hospital. Due to its retrospective nature and the anonymous nature of data collection, our study was exempted from the need for written informed consent by the Ethics Committee of Shandong Provincial Chest Hospital.

## Author Contributions

M-SW and Y-AZ designed the study and supervised data collection. H-RW, CH, and J-LW collected the data, performed statistical analysis, and drafted the initial manuscript. All authors approved the final version of the report.

## Funding

This work was supported by the Science Research and Technology Development Plan of Baise City (20203405).

## Conflict of Interest

The authors declare that the research was conducted in the absence of any commercial or financial relationships that could be construed as a potential conflict of interest.

## Publisher's Note

All claims expressed in this article are solely those of the authors and do not necessarily represent those of their affiliated organizations, or those of the publisher, the editors and the reviewers. Any product that may be evaluated in this article, or claim that may be made by its manufacturer, is not guaranteed or endorsed by the publisher.
